# Is a Dissociation Process Underlying the Molecular Origin of the Debye Process in Monohydroxy Alcohols?

**DOI:** 10.1021/acs.jpcb.0c10970

**Published:** 2021-03-11

**Authors:** N. Soszka, B. Hachuła, M. Tarnacka, E. Kaminska, S. Pawlus, K. Kaminski, M. Paluch

**Affiliations:** † Institute of Chemistry, University of Silesia in Katowice, Szkolna 9, 40-006 Katowice, Poland; ‡ August Chełkowski Institute of Physics, University of Silesia in Katowice, 75 Pulku Piechoty 1, 41-500 Chorzow, Poland; § Silesian Center for Education and Interdisciplinary Research, 75 Pulku Piechoty 1a, 41-500 Chorzow, Poland; ∥ Department of Pharmacognosy and Phytochemistry, Faculty of Pharmaceutical Sciences in Sosnowiec, Medical University of Silesia in Katowice, ul. Jagiellońska 4, 41-200 Sosnowiec, Poland

## Abstract

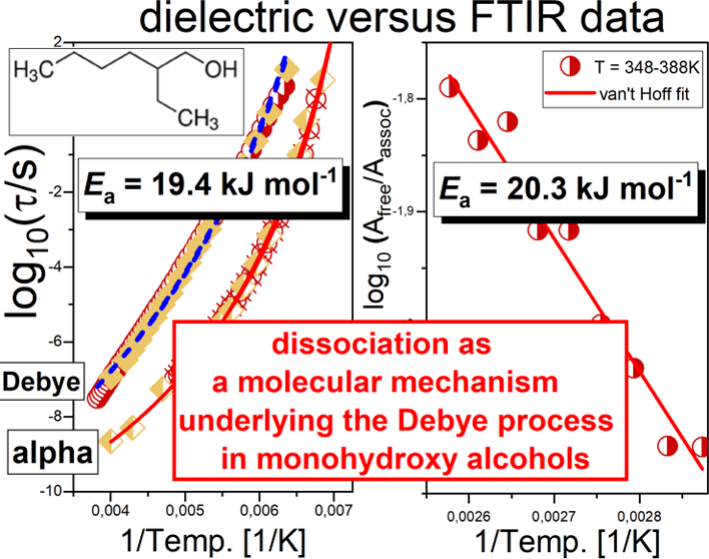

Herein, we investigated the molecular dynamics as well as intramolecular interactions in two primary monohydroxy alcohols (MA), 2-ethyl-1-hexanol (2EHOH) and *n*-butanol (*n*BOH), by means of broad-band dielectric (BDS) and Fourier transform infrared (FTIR) spectroscopy. The modeling data obtained from dielectric studies within the Rubinstein approach [Macromolecules2013, 46, 7525−7541] originally developed to describe the dynamical properties of self-assembling macromolecules allowed us to calculate the energy barrier (*E*_a_) of dissociation from the temperature dependences of relaxation times of Debye and structural processes. We found *E*_a_ ∼ 19.4 ± 0.8 and 5.3 ± 0.4 kJ/mol for the former and latter systems, respectively. On the other hand, FTIR data analyzed within the van’t Hoff relationship yielded the energy barriers for dissociation *E*_a_ ∼ 20.3 ± 2.1 and 12.4 ± 1.6 kJ/mol for 2EHOH and *n*BOH, respectively. Hence, there was almost a perfect agreement between the values of *E*_a_ estimated from dielectric and FTIR studies for the 2EHOH, while some notable discrepancy was noted for the second alcohol. A quite significant difference in the activation barrier of dissociation indicates that there are probably supramolecular clusters of varying geometry or a ring-chain-like equilibrium is strongly affected in both alcohols. Nevertheless, our analysis showed that the association/dissociation processes undergoing within nanoassociates are one of the main factors underlying the molecular origin of the Debye process, supporting the transient chain model.

## 1 Introduction

Systematic, long-term investigations on the dynamics of supercooled glass-forming materials revealed that in the vast majority of cases, the shape of relaxation processes (including structural, α, one) is clearly stretched exponential independently on the used spectroscopic method.^1−3^ In fact, this experimental finding, which is still yet to be clarified, became a universal feature of systems approaching the glass-transition temperature, *T*_g_. However, for certain associating liquids, the exponential decay of the electric polarization (called the Debye, *D*, relaxation), characterized by a single time constant, has been detected.^4^ Such a specific mode is especially well visible in monohydroxy alcohols (MA) having hydroxyl unit(s) attached to the terminal carbon. In these materials, the *D*-process dominates the dielectric response.^5,6^ Consequently, the structural *α*-relaxation is frequently manifested only as an excess wing on the *D*-peak’s high-frequency flank. Due to the unique character of the Debye relaxation, great effort has been made to understand its molecular mechanism. In the literature, several, sometimes contradictory, possible explanations of this process have been given. Herein, we only list a few of them, i.e., the connection between the *D*-process and proton conductivity within the reverse micelles according to Maxwell–Wagner–Sillars effects in heterogeneous dielectrics,^7^ breaking and forming new H-bonds between neighbor molecules, a lifetime of these specific interactions,^8,9^ or fluctuations of the end-to-end vector of associated multimers.^10^ More recently, this characteristic relaxation has been assigned to the dipole–dipole interactions,^11^ a migration of defects through the H-bonded network,^12^ or the transition from the ring to the linear architecture of supramolecular structures as deduced from dielectric measurements carried out in the nonlinear regime.^13^ However, from all of the hypotheses mentioned above, the transient chain model (TCM) proposed by Gainaru et al.,^14^ considering the continuous attachment and detachment of the single MA molecules to the linear nanoassociates triggering dipole moment and, consequently, the polarization variation, provides by far the best description of the dynamical properties of the *D*-mode. In addition, this concept was supported by the results of rheological investigations.^15^ It was found that aside from the structural process, there is also an additional relaxation mode (mimicking the terminal relaxation in polymers) in the shear modulus spectra in some primary alcohols. Interestingly, the time scale of this process in rheological response was comparable to that observed in dielectric loss spectra. Nevertheless, it should be mentioned that although the TCM seems to be quite a plausible approach, it has never been verified experimentally. Even a combination of the data coming from different experimental techniques, such as Fourier transform infrared (FTIR), broad-band dielectric (BDS) and nuclear magnetic resonance (NMR) spectroscopy, dynamic mechanical and thermal analysis (DMTA), or dynamic light scattering (DLS), did not allow us to unquestionably certify that the TCM model properly explains the molecular origin of the *D*-process in MA. It is worth pointing out that understanding the real mechanism of the *D*-mode becomes even more crucial due to an increasing number of papers reporting the presence of this kind of mobility in other liquids tending to associate via H-bonds or van der Waals interactions.^16−20^ Furthermore, solving this fundamental puzzle seems to be a crucial step to gain a unique insight into the behavior or properties of H-bonds in materials subjected to various external conditions, such as temperature, pressure, high electric field, solutions, etc.

In this work, we have studied two primary MA, *n*-butanol (1-butanol, *n*BOH) and 2-ethyl-1-hexanol (2EHOH), characterized by a significant separation between time scales of structural and Debye processes. We have used BDS and FTIR spectroscopy techniques, which allowed us to show that the dissociation process is an important factor responsible for the *D*-relaxation. Furthermore, we have estimated the activation barrier, *E*_a_, of this phenomenon using dielectric data implementing the Rubinstein approach,^21^ derived originally for self-assembled polymers. The obtained values agree well with *E*_a_ of the dissociation process determined from the FTIR investigations. It is also worth adding that the presented spectroscopic results extend and complement the data published in the previous papers,^22−26^ creating a more thorough picture of the behavior of self-assembling alcohols during the dissociation process.

## 2 Experimental Section

### 2.1 Materials

**Figure 1:**
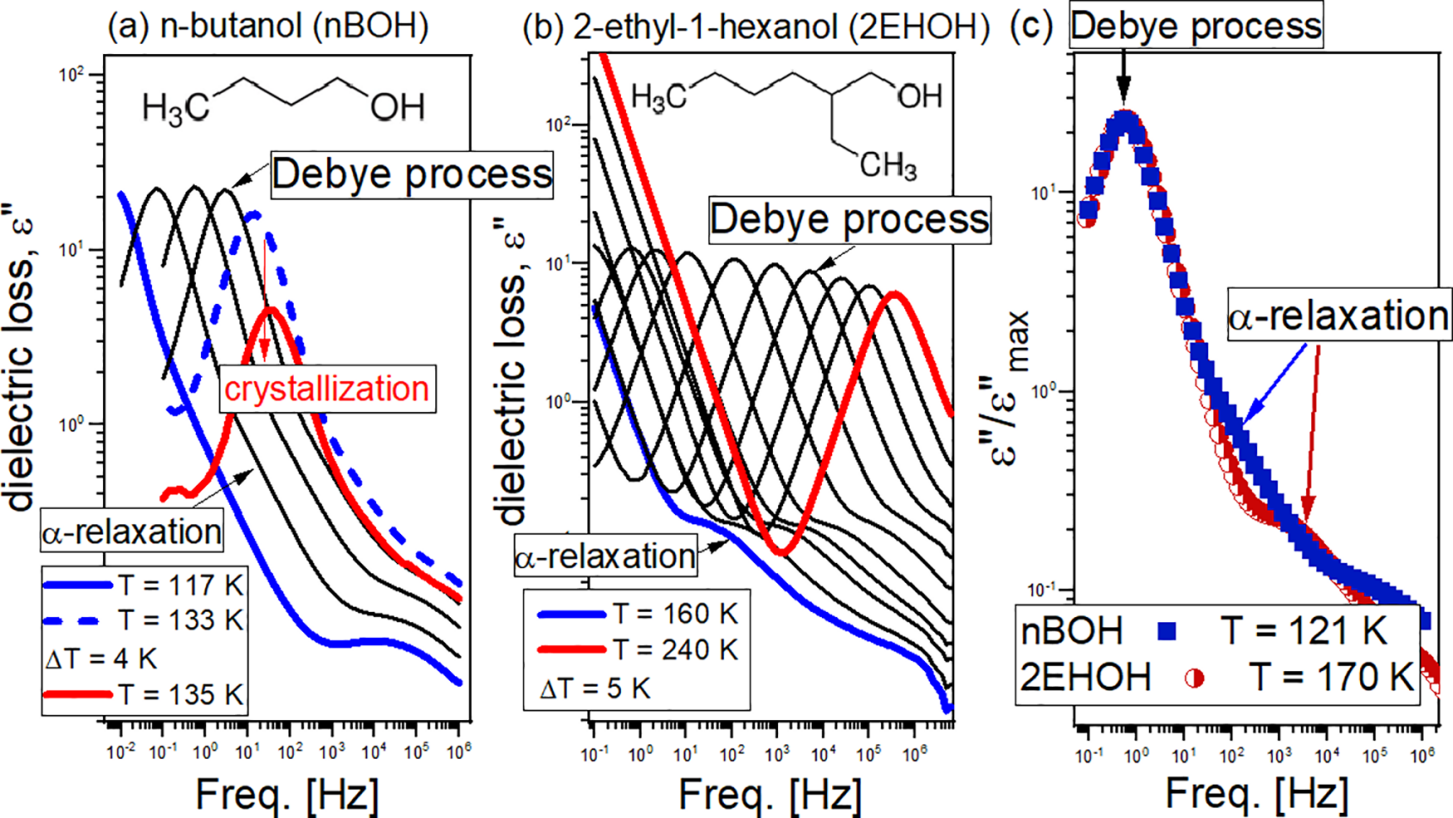
(a, b) Dielectric loss spectra of *n*-butanol (*n*BOH) and 2-ethyl-1-hexanol (2EHOH) measured above their glass-transition temperatures, *T*_g_. A decreasing amplitude of both *D*- and α-processes observed in *n*BOH with increasing temperature indicates the ongoing crystallization or a formation of glacial phase, which explains a limited set of dielectric data recorded for this compound. The insets in (a) and (b) show the chemical structures of the investigated compounds. (c) Comparison of dielectric loss spectra at a constant Debye relaxation time, τ_D_, near *T*_g_.

2-Ethyl-1-hexanol and *n*-butanol of purity higher than 99% were supplied from Sigma-Aldrich and used as received. The chemical structures of the investigated alcohols are presented in the insets to Figure 1.

### 2.2 Methods

#### 2.2.1 Broad-Band Dielectric Spectroscopy

BDS measurements were carried out on heating after a fast quenching of liquid samples, in a wide range of temperatures (120–298 K) and frequencies (10^–1^–10^6^ Hz), using a Novocontrol spectrometer, equipped with an Alpha impedance analyzer, an active sample cell, and Quatro Cryosystem. The capacitor was built from two stainless steel electrodes (15 mm diameter), distanced with two 100 μm thick glass fibers, and sealed within a Teflon ring.

#### 2.2.2 Fourier Transform Infrared Spectroscopy

An FTIR Thermo Scientific Nicolet iS50 spectrometer was used to measure the studied alcohols’ infrared absorption spectra at room temperature (RT = 293 K) and *T*_g_. A total of 16 scans were collected from 800 to 4000 cm^–1^ with a spectral resolution of 2 cm^–1^. During the experiments, liquid nitrogen was flowed into the FTIR spectrometer to avoid atmospheric H_2_O and CO_2_ interference in the spectrum. The liquid cell equipped with two CaF_2_ windows, separated by a 15 μm thick spacer, was used to obtain the sample film of uniform thickness and guarantee the system’s constant geometry. The cell was placed into a Linkam Scientific Instruments THMS 600 heating/cooling stage, which allowed cooling the samples to *T*_g_. The cooling rate was 10 K/min. The temperature stabilization accuracy was 0.1 K. High-temperature FTIR spectra were obtained using a Nicolet iS50 FTIR spectrometer (Thermo Scientific) coupled with GladiATR (Pike Technologies) with a single reflection monolithic diamond. They were collected in the range of 4000–400 cm^–1^ at a resolution of 2 cm^–1^. For every spectrum, 16 scans were averaged. The absorption signals were measured every 2 K (*n*BOH) or 5 K (2EHOH) from *T* = 298 to 388 K (see Figure S1). Background spectra were recorded before the sample measurements and were used for background correction. Both low- and high-temperature FTIR spectra were collected from room temperature, without any additional thermal treatment. MagicPlot software (version 2.9.3, MagicPlot Systems LLC, Saint Petersburg, Russia) was used to perform the OH stretching bands’ deconvolution process. The decomposition of this band into separate components involved the following three steps: (i) curve fitting of the band occurring between 3050 and 3800 cm^–1^, carried out with the use of two Gaussian functions adjusting the intensity and the width of the curves; (ii) subtracting the fitted spectrum of the H-bonded OH band from the original IR spectra; and (iii) peak fitting of the free OH band occurring between 3600 and 3650 cm^–1^ (Figure S2). It was important to accurately determine the H-bonded OH band’s spectral parameters since it strongly affected the free OH band’s final intensities and bandwidths. Both free and H-bonded OH band components were not involved in the decomposition together because a large error is obtained during fitting of a poorly resolved broad OH band with multiple components. In addition, we used the described deconvolution procedure to remove the contribution of H-bonded OH groups from the free hydroxyl groups. In this way, we obtained a better fitting of the peak of free OH groups occurring above the 3600 cm^–1^ frequency range. We did not carry out the decomposition of the OH bands for the samples measured below 348 K (2EHOH) and 358 K (*n*BOH) since the free OH groups’ peak was hardly detectable. Consequently, the values of peak areas were within the limits of experimental error. It was related to much stronger interactions between molecules of the examined MA at lower temperatures. All spectral parameters were left free during the fitting procedure.

## 3 Results and Discussion

Figure 1a,b presents dielectric loss spectra of both MA measured above their glass-transition temperatures, *T*_g_. The data reveal multiple relaxation processes: starting from the low frequencies (i) the dc conductivity (connected to the charge transport of ions), (ii) the previously mentioned Debye mode,^27,28^ and (iii) the structural α-relaxation process (responsible for the cooperative motions of molecules and the glass transition). Further temperature decrease leads to the appearance of secondary β-relaxation at higher frequencies. It is worth emphasizing that, above *T*_g_, the recorded dielectric spectra are dominated by the prominent *D*-process, which has a significantly larger (by more than 1 order of magnitude) amplitude compared to the α-mode. Consequently, α-relaxation is detected only as an excess wing on the high-frequency flank of the *D*-process. It is worthwhile to stress that the structural process is much better resolved from the *D*-mode in 2EHOH with respect to *n*BOH (Figure 1c).

To gain a better insight into the possible molecular origin of *D*-relaxation in monohydroxy alcohols, we applied the Rubinstein approach derived initially to describe the properties of various supramolecular macromolecules with a different number of self-associating groups.^21^ One can briefly remind that for polymers, dielectric loss spectra measured above *T*_g_ also revealed the presence of an additional relaxation mode (slower than the α-process).^21,29,30^ This additional mode (labeled as α*) was assigned to the dissociation between functional moieties, acting as stickers, with the relaxation times, τ_α*_, interpreted as “the mean lifetime of the associated state”.^31,32^ According to this approach, the activation barrier of dissociation/association (*E*_a_, necessary to break or form H-bonds) can be estimated from the temperature dependences of τ_α*_ and τ_α_ (structural/segmental relaxation times) as follows
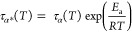
1where *R* is the gas constant.^32,33^

**Figure 2:**
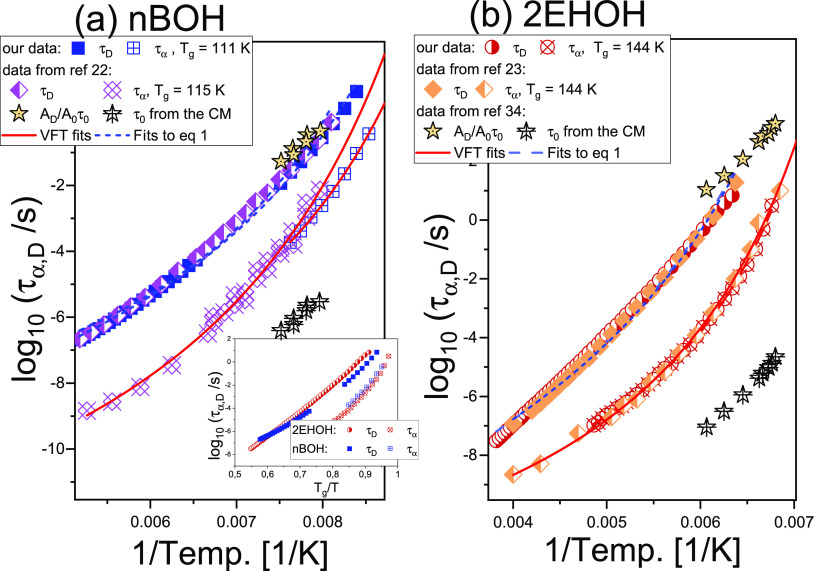
Temperature dependences of structural, τ_α_, and Debye, τ_D_, relaxation times determined for both *n*BOH (a) and 2EHOH (b) plotted together with the literature data taken from refs (22, 23). In addition, we also included temperature dependences of primitive relaxation times calculated from the coupling model, CM (open star symbols) multiplied by *A*_D_/*A*_0_ factor (filled stars) as discussed in ref (34). The inset in (a) shows the τ(*T*) dependence plotted as a function of *T*_g_/*T*. The solid red and dashed blue lines are the best fits of τ_α_(*T*) dependences to the VFT equation (eq 2) and *τ*_D_(*T*)-dependences to eq 1, respectively.

Hence, considering the similarities between the Debye process and the α*-mode reported for various associating polymers, we had modified eq 1 assuming τ_α*_ ∼ τ_D_. All τ(*T*) dependences calculated from the fitting analysis of the data shown in Figure 1 with the superposition of two (or three) Havriliak–Negami (HN) functions with an additional dc conductivity term^35^ are shown in Figure 2 together with the literature data taken from refs (22, 23). Representative HN fits and the corresponding fitting parameters are presented in Figure S3 and Table S1. According to the approach reported in ref (31), first, τ_α_(*T*) dependences were fitted to the Vogel–Fulcher–Tammann (VFT) equation^36−38^
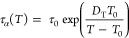
2where τ_0_ is the relaxation time at finite temperature, *D*_T_ is the strength parameter, and *T*_0_ represents the temperature at which structural relaxation times tend to infinity; see solid red lines in Figure 2. The VFT fit parameters obtained from the fitting our data and those taken from the literature are listed in Tables 1 and S2, respectively. Additionally, in Table 1, we presented values of the glass-transition temperatures, *T*_g_ (defined as a temperature at which τ_α_ = 100 s), determined from dielectric data for both investigated compounds. One can recall that the calculated *T*_g_s agree perfectly with those reported in refs (22, 23). In addition, τ(*T*) dependences plotted as a function of *T*_g_/*T* are shown as an inset in Figure 2a.

**Table 1:** VFT Fit Parameters of the α-Process, Glass-Transition Temperatures Determined from eq 2, and Activation Barriers of Dissociation Calculated According to Eq 1

sample	log (τ_0_ [s])	*D*_T_	*T*_0_ [K]	*T*_g_ [K] for τ_α_ = 100 s	*E*_a_ [kJ/mol]
2EHOH	–11.06	862.5	115.0	144	19.4 ± 0.8
*n*BOH	–10.86	730.4	86.8	111	5.3 ± 0.4

Next, τ_D_(*T*) dependences were fitted to eq 1 using the VFT fit parameters of the respective α-peak, where the activation barrier of dissociation, *E*_a_, was the only fitting parameter. As observed in Figure 2, the dashed blue lines, which are the best fits to eq 1, describe τ_D_(*T*) dependencies quite well. Interestingly, this analysis yields the activation barrier of dissociation, *E*_a,*n*BOH_ = 5.3 ± 0.4 kJ/mol and *E*_a,2EHOH_ = 19.4 ± 0.8 kJ/mol. However, utilizing VFT fits of structural relaxation times determined in a wider range of temperatures (data taken from refs (22, 23)), we got the activation barrier for dissociation process equal to *E*_a,*n*BOH_ = −5.1 ± 0.3 kJ/mol and *E*_a,2EHOH_ = 13.5 ± 1.0 kJ/mol; see Figure 2. Although in the case of 2EHOH, *E*_a_ was still positive, it reaches a negative value for *n*BOH. The determined values of *E*_a_ and the VFT parameters obtained for τ_α_(*T*) dependencies are listed in Table S2. One can suppose that a negative activation barrier might be a signature of more associative-type processes undergoing in the latter alcohols. However, one has to be careful interpreting this finding due to the presence of crossover temperature^28^ or the existence of the glacial phase in n-butanol at higher temperatures.^39^ One can expect that formation of this new state, which is considered as either a manifestation of polyamorphism in liquids or, alternatively, frustrated or aborted crystallization process that produces plenty of nanocrystallites of the stable crystalline phase embedded in a disordered matrix,^40−43^ might have a strong influence on the evolution of structural relaxation times and an outcome of the analysis using the Rubinstein model. Finally, it should be pointed out that this approach was originally derived to describe self-assembly processes in polymers, not low-molecular-weight glass formers that form extensive H-bonded nanoassociates. In this context, it is worth noting that supramolecular clusters mimic the polymer behavior only in the close vicinity of the glass-transition temperature, where H bonds are strong, and associates are the most physically stable. One can mention that in ref (34), the authors have presented a test to verify whether the formed chains are stable and the structural process resembles the segmental relaxation of a polymer. For that purpose, they applied the coupling model (CM), where the calculated primitive relaxation times were multiplied by the factor *A*_D_/*A*_0_ to superpose with the ones determined for the Debye process. It was found that a close agreement between *A*_D_/*A*_0_τ_0_ and τ_D_ was found only around the glass-transition temperature (see filled stars in Figure 2b) in 2EHOH, n-propanol, or 5-methyl-2-hexanol. A similar procedure was also applied in *n*BOH (for which the stretching parameter reaches β_KWW_ = 0.54, or *n* = 0.46), where the same scenario was noted (see filled stars in Figure 2a). This probably means that the most valuable information on the dissociation (or association) processes occurring in alcohols can be obtained from fitting the data measured in the vicinity of *T*_g_ to the Rubinstein model. Herein, it should also be added that in hydroxyl-terminated poly(propylene glycol) and poly(dimethylsiloxane) derivatives, *E*_a_ ∼ 5.8–9.1 kJ/mol,^31^ whereas for urazole-functionalized entangled polyisoprene and poly(mercaptopropyl)methylsiloxane, *E*_a_ ∼ 25–29 kJ/mol.^32,44^

Having the values of *E*_a_ estimated from dielectric data for both examined MA, we decided to compare them to the respective ones calculated from FTIR investigations. One can assume that the agreement between *E*_a_ determined from both experimental techniques could be further evidence that the appearance of the *D*-process is related to the continuous dissociation/association between MA molecules creating longer self-assemblies (of approximately “supramolecular” structures) and affecting the dipole moment (as well as the polarization), as proposed by the TCM approach.

**Figure 3:**
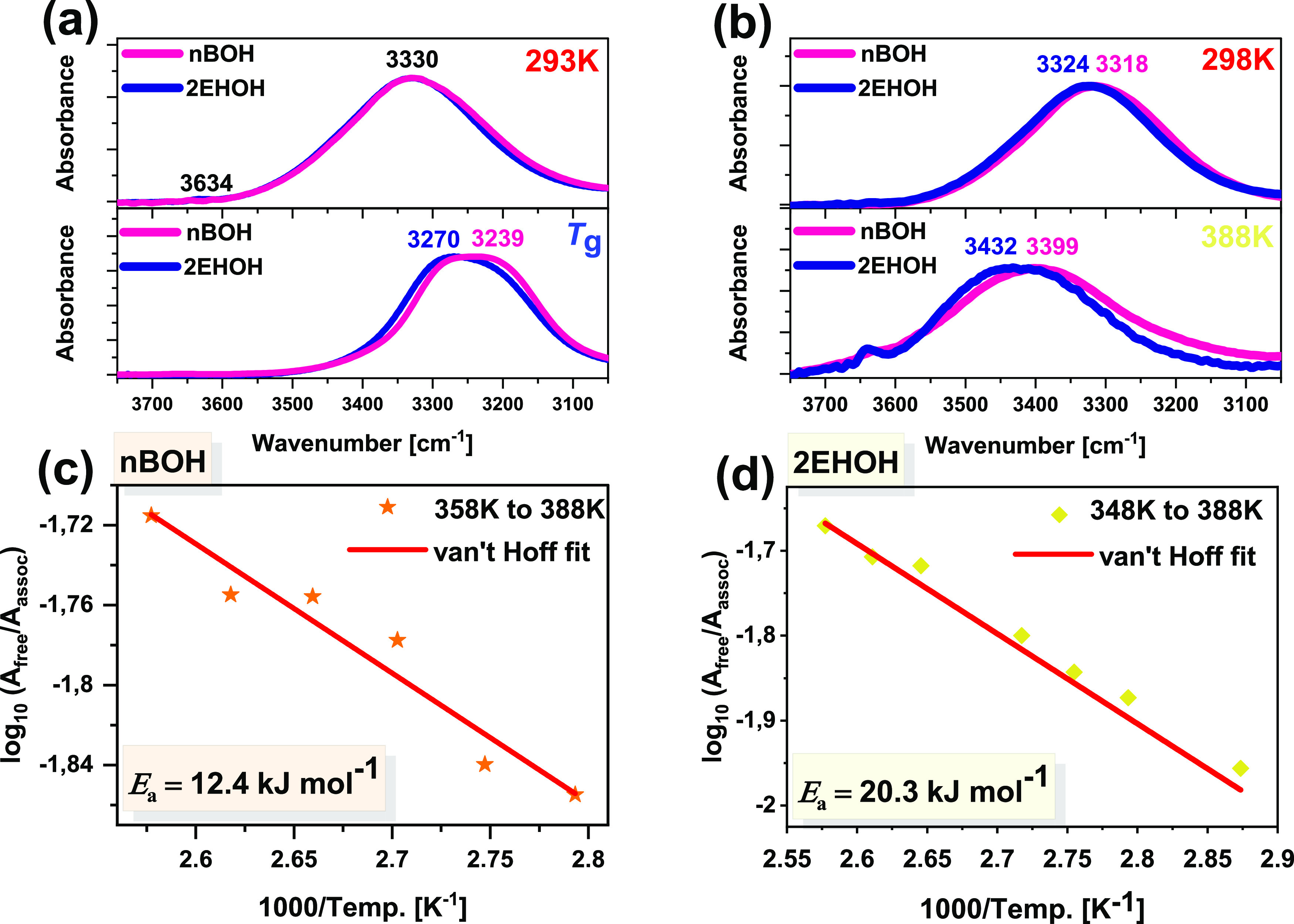
FTIR spectra of *n*BOH and 2EHOH in the spectral region between 3750 and 3050 cm^–1^ measured at (a) *T* = 293 K and *T*_g_ and (b) *T* = 298 K and *T* = 388 K. The spectra were normalized to the OH stretching vibration peak intensity. (c, d) van’t Hoff plots for the IR absorption OH bands of *n*BOH and 2EHOH used to derive the dissociation enthalpy between the free and H-bonded OH species.

However, before we focus our attention to determine the activation energy for the dissociation in *n*BOH and 2EHOH, first, the properties of H-bonds in both systems will be discussed. Figure 3a presents the IR spectra of alcohols in the OH vibrations’ spectral range at room temperature (RT = 293 K) and *T*_g_. The high-frequency region covering the range of about 3000–3700 cm^–1^ is dominated by an intense, broad band attributed to the OH stretching vibrations. Generally, the OH groups forming H-bonds absorb strongly below 3600 cm^–1^, whereas the band arising from the non-H-bonded “free” OH groups occurs above this wavenumber. Comparing the spectral profile of the OH stretching band of both alcohols at RT, one can see that their ν_OH_ band appears at the same frequency position (3300 cm^–1^). It is notable that the H-bonded ν_OH_ peak for *n*BOH is slightly broader than 2EHOH, i.e., the ν_OH_ bandwidths (the full width at half-maximum, FWHM) are 231.51 cm^–1^ vs 224.56 cm^–1^, respectively. It suggests a greater heterogeneity in the H-bonding network for this compound. Surprisingly, at *T*_g_, the ν_OH_ bandwidths for both alcohols become more comparable (190.40 cm^–1^ for *n*BOH and 195.18 cm^–1^ for 2EHOH). After cooling, the shift of the OH stretching vibration band to lower wavenumbers occurs (Δν), i.e., the ν_OH_ peak appears at 3239 and 3270 cm^–1^ for *n*BOH and 2EHOH (Δν = 92 cm^–1^ for *n*BOH and Δν = 60 cm^–1^ for 2EHOH). It is worth mentioning that in the case of 2EHOH, a weak peak at 3634 cm^–1^ is detected at RT, indicating the presence of a small amount of free OH groups in this system. As the temperature decreases, this peak disappears. In the case of *n*BOH, no peak above 3600 cm^–1^ is clearly visible at both studied temperatures, which means that almost all OH groups are hydrogen-bonded.

In the next step, the FTIR spectra of *n*BOH and 2EHOH were measured as a function of temperature in the range *T* = 298–388 K to determine the equilibrium processes between H-bonded and free MA, as well as to calculate the energy barrier for dissociation from the van’t Hoff relationship
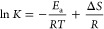
3where *E*_a_ and Δ*S* are the activation enthalpy and entropy of the dissociation process, respectively.

The representative spectra obtained at *T* = 298 and 388 K, in the OH stretching frequency range, are shown in Figure 3b. Figure S1 presents the temperature evolution of IR spectra of alcohols under investigation during the heating process. It is observed that the high-frequency region (3700–3000 cm^–1^) of the analyzed IR spectra contains a strong, broad band of the stretching vibrations of OH groups, ν_OH_, which is adjacent to the C–H stretching vibrations (3000–2800 cm^–1^). At RT, the intensity maximum of the ν_OH_ band for *n*BOH occurs at a lower frequency than 2EHOH (3318 cm^–1^ vs 3324 cm^–1^; see Table S3). It means that intermolecular H-bonds of *n*BOH are slightly stronger than those found in 2EHOH at *T* = 298 K (Figure 3b). A similar pattern of behavior is also observed at *T* = 388 K (3399 cm^–1^ vs 3432 cm^–1^). It can be seen that the band position occurring ca. 3320 cm^–1^ at *T* = 298 K, assigned to the H-bonded OH groups, shifts to a higher frequency (Δν = 81 cm^–1^ for *n*BOH, Δν = 108 cm^–1^ for 2EHOH; Figure S1) with increasing temperature. It is related to the weakening of H-bonding interactions. Simultaneously, the intensity of this band notably decreases, which is attributed to the change in the absorption coefficient as the populations and strength of the H bonds are reduced. Interestingly, the ν_OH_ band intensity decreases after a temperature rise is observed to a different extent for both alcohols (by around 37% for *n*BOH and 60% for 2EHOH). Furthermore, the bandwidth of the OH stretching vibration consistently grows upon heating. Such behavior is related to the increase of the heterogeneity in the H-bonding network at higher temperatures. On analyzing IR spectra of both alcohols in the higher-frequency range, one can observe a very weak peak occurring above 3600 cm^–1^. This absorption signal corresponds to the existence of non-hydrogen-bonded free OH groups. At *T* = 298 K, the contour of this band is barely notable for 2EHOH, while in *n*BOH, it is not detectable. As the temperature increases, the intensity of the free OH band increases, indicating the H-bond breaking in both systems. It was also observed that the free ν_OH_ band has a higher intensity for 2EHOH in comparison to *n*BOH at *T* = 388 K, which is an indication of the lower degree of association of this alcohol.

Using the IR experiment’s integrated intensity values, one can calculate the formation/breaking enthalpy of H-bonded hydroxyl groups. It is done by examining the equilibrium between the free OH and H-bonded OH species at a given temperature. Note that for temperatures lower than *T* = 348 K, the position of the free ν_OH_ band becomes erratic due to significant (background) noise. Thus, the data measured at temperatures lower than *T* = 348 K were not considered during further calculations. To obtain the integrated absorbance values of the free and H-bonded OH bands at measured temperatures (that can be linked to the concentration of both populations of MA), the deconvolution of these bands using MagicPlot software was performed. The ratio *A*_free_/*A*_assoc_ was found to increase monotonically with increasing temperature from *T* = 348 to 388 K. Next, the van’t Hoff plots (the dependence of log *K* vs 1/*T*) shown in Figure 3c,d were constructed. Fitting the data to eq 3 yields *E*_a,*n*BOH_ = 12.4 ± 1.6 kJ/mol and *E*_a,2EHOH_ = 20.3 ± 2.1 kJ/mol. Those values correspond to the enthalpy change for the dissociation process  between the H-bonded and free O–H species in *n*BOH and 2EHOH, respectively. The positive *E*_a_ means that a temperature-induced H-bonding dissociation process is occurring in both alcohols. It is notable that *n*BOH exhibits lower dissociation enthalpy, which indicates that less energy is needed to break H-bonds in this alcohol compared to 2EHOH. It is also worth stressing that from the same FTIR dataset, the association’s energy can also be estimated, which is essentially equal to *E*_a_ of dissociation but has a negative sign. The difference in the activation energies of the H-bond dissociation in both alcohols is a quite interesting finding, considering that 2EHOH and *n*BOH are first-order alcohols, differing only in the length of the alkyl chain and steric hindrance. Based on the literature data, one can conclude that both alcohols tend to form rather chainlike oligomeric self-assemblies consisted of around 10 molecules.^42,45^ However, previous dielectric studies indicated that they differ in the value of the Kirkwood factor (*g*_k_ ∼ 3 and *g*_k_ ∼ 4 around *T*_g_ for 2EHOH and *n*BOH, respectively^25,46^), which defines the long-distance correlation between dipoles. It means that steric hindrance plays an important role and may affect the architecture of supramolecular associates as well as a chain-ring equilibrium in both alcohols. Furthermore, the ethyl C–H bonds may stabilize the supramolecular structure of 2EHOH via the formation of additional weaker C–H···O interactions. As a result, the dissociation of the H-bonded network in 2EHOH may require more energy than the dissociation of the chainlike system of *n*BOH.

Herein, it should also be stressed that the values of *E*_a_ obtained by us using the van’t Hoff relation are not consistent with those given by Bauer et al.^24^ This is due to the different methods of experimental data analysis. Namely, Bauer et al.^24^ analyzed the bands of free and associated OH groups based on the peak intensity (absorbance) values in the hydroxyl region of near-infrared (NIR) spectra. In turn, we carried out the decomposition of the ν_OH_ bands based on the peak area of free and H-bonded OH groups. Moreover, this fitting was performed in three stages/steps, which allowed us to separate the signal of free OH groups (located on the high-energy wing of H-bonded OH band) from the associated OH groups. Moreover, it was shown that for different MA, the value of enthalpy changes nonlinearly with decreasing temperature.^24^ According to Hédoux et al.,^47^ this experimental fact may be interpreted as resulting from the competition between the development of a preferred local organization at a low temperature, characterized by molecular associations via strong H-bonds and the extension of the intermediate-range order characterized by molecular associations bonded by weaker interactions.

Based on the comparison of the activation barrier for dissociation estimated from the analysis of dielectric data using the Rubinstein model and FTIR data, one can claim that there is a good agreement between them in the case of 2EHOH. At the same time, some notable discrepancy is noted for *n*BOH. One can argue that this discrepancy originates from a strong contribution to the temperature evolution of the structural process and its possible connection to H-bond dynamics in both alcohols. In this context, it is worth recalling that Gainaru et al.^23^ have shown that the reorganization of H-bonds in 2EHOH occurs at the time scale of the structural process. Moreover, it should be emphasized that due to the enhanced crystallization or formation of the glacial phase in *n*BOH, τ_α_(*T*) dependence in this alcohol might be strongly affected, leading to a negative activation barrier for dissociation. Both the above-described reasons could contribute to the discrepancy between the activation barrier for dissociation calculated from both experimental methods. One can also argue that the lack of perfect agreement between both activation barriers determined from dielectric and infrared data is an evidence that the dissociation process, although important, is not the only factor responsible for the appearance of the Debye process in dielectric loss spectra. Nevertheless, it is worth pointing out that the variation in trend in both quantities is well preserved. Hence, one can emphasize that the Rubinstein approach^21^ provides a quite interesting insight into the variation in the MA’s dissociation energy and offers a unique opportunity to understand the molecular origin of the Debye process, supporting the TCM model proposed by Gainaru et al.^14^

## 4 Conclusions

In this work, we applied the Rubinstein approach initially developed to determine the self-assembling properties of macromolecules, to model dielectric data obtained for the two low-molecular-weight H-bonded primary MA. Interestingly, a good correspondence between the activation energy of dissociation determined from dielectric and FTIR data for 2EHOH and some discrepancy between both energy barriers in the case of *n*BOH were found. Moreover, fitting the data in a broader range of temperatures (data taken from ref (22)) yields negative activation energy for dissociation. This might indicate the associative type of processes dominating in this alcohol. Nevertheless, as discussed in the main text, one has to be careful in the interpretation of this finding since (i) the Rubinstein model was developed to describe the self-assembly in polymers, (ii) *n*BOH forms glacial phase at higher temperatures, and (iii) the stability of nano-self-assemblies mimicking the behavior of polymers is enhanced only in the vicinity of the glass-transition temperature. All of these factors might affect the temperature evolution of relaxation times of structural and Debye processes and the outcome of the dielectric data analysis. At the moment, it is highly required to combine dielectric and FTIR data obtained for different kinds of alcohols to see, whether there will be any correlation between *E*_a_ estimated from infrared studies and the Rubinstein approach. It must also be stressed that the analysis performed in this paper demonstrated that *E*_a_ of 2EHOH is much higher with respect to *n*BOH. This probably indicates a change in the architecture of supramolecular clusters or variation in the ring-chain equilibrium in both alcohols. Alternatively, one can suppose that the ethyl C–H bonds may stabilize the supramolecular structure of 2EHOH via formation of additional weaker C–H···O interactions, contributing to the higher energy required to break H bonds. Finally, the lack of perfect agreement between activation barriers for dissociation calculated from dielectric and infrared data can be an evidence that the dissociation process, although important, is not the only factor responsible for the appearance of the Debye process in dielectric loss spectra.

## Supporting Information Available

Fit of dielectric spectra with the use of two HN functions and the determined relaxation times; temperature-dependent FTIR spectra with the spectral parameters at 298 and 388 K; and decomposition of ν_OH_ bands under various temperature conditions (PDF)
