# Supplementary material for: Is a Dissociation Process Underlying the Molecular Origin of the Debye Process in Monohydroxy Alcohols?
Source: J Phys Chem B. 2021 Mar 11;125(11):2960–7. doi: 10.1021/acs.jpcb.0c10970 (PMC8041310; doi:10.1021/acs.jpcb.0c10970)
Supplement: Supplementary file 1 — jp0c10970_si_001.pdf [file jp0c10970_si_001.pdf]

Supporting Information

Is a Dissociation Process Underlying the Molecular Origin of the Debye Process in Monohydroxy Alcohols?

N. Soszka[1,2], B. Hachuła[1,3*], M. Tarnacka[2,3], E. Kaminska[4], S. Pawlus[2,3],

K. Kaminski[2,3*], M. Paluch[2,3]

[1] Institute of Chemistry, University of Silesia in Katowice, Szkolna 9, 40-006 Katowice, Poland
[2] August Chełkowski Institute of Physics, University of Silesia in Katowice, 75 Pulku Piechoty 1, 41-500 Chorzow, Poland
[3] Silesian Center for Education and Interdisciplinary Research, 75 Pulku Piechoty 1a, 41-500 Chorzow, Poland
[4] Department of Pharmacognosy and Phytochemistry, Faculty of Pharmaceutical Sciences in Sosnowiec, Medical University of Silesia in Katowice, ul. Jagiellońska 4, 41-200 Sosnowiec, Poland

*Corresponding authors: (B.H.) barbara.hachula@us.edu.pl; (K.K.) kamil.kaminski@us.edu.pl; kamil.kaminski@smcebi.edu.pl

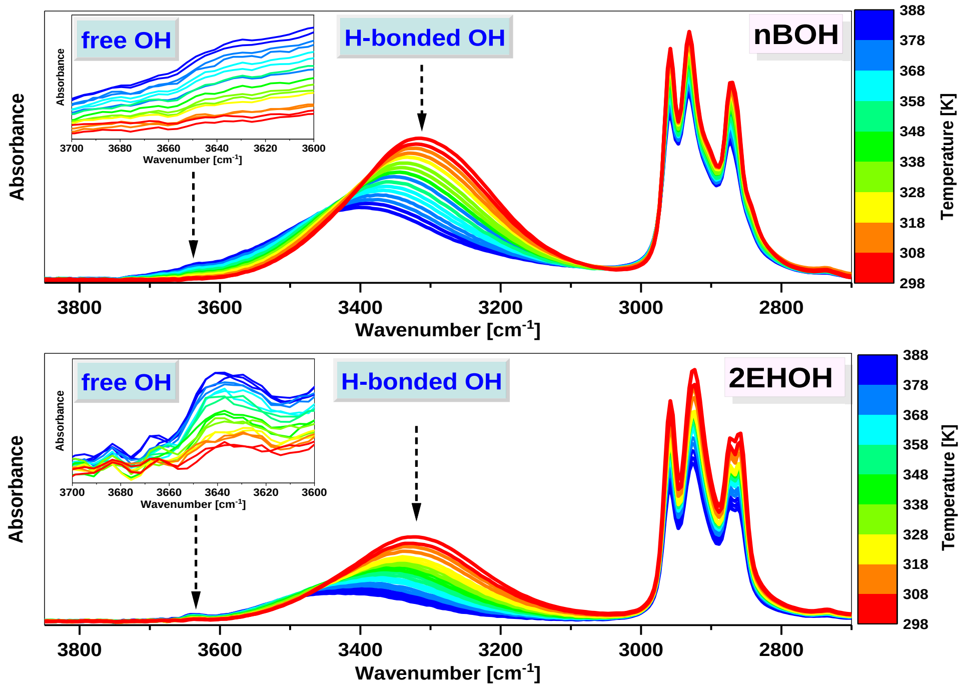

Figure S1. Temperature-dependent FTIR spectra of nBOH and 2EHOH measured at temperatures *T* = 298 – 388 K in the frequency range of 3850-2700 cm^{-1}.

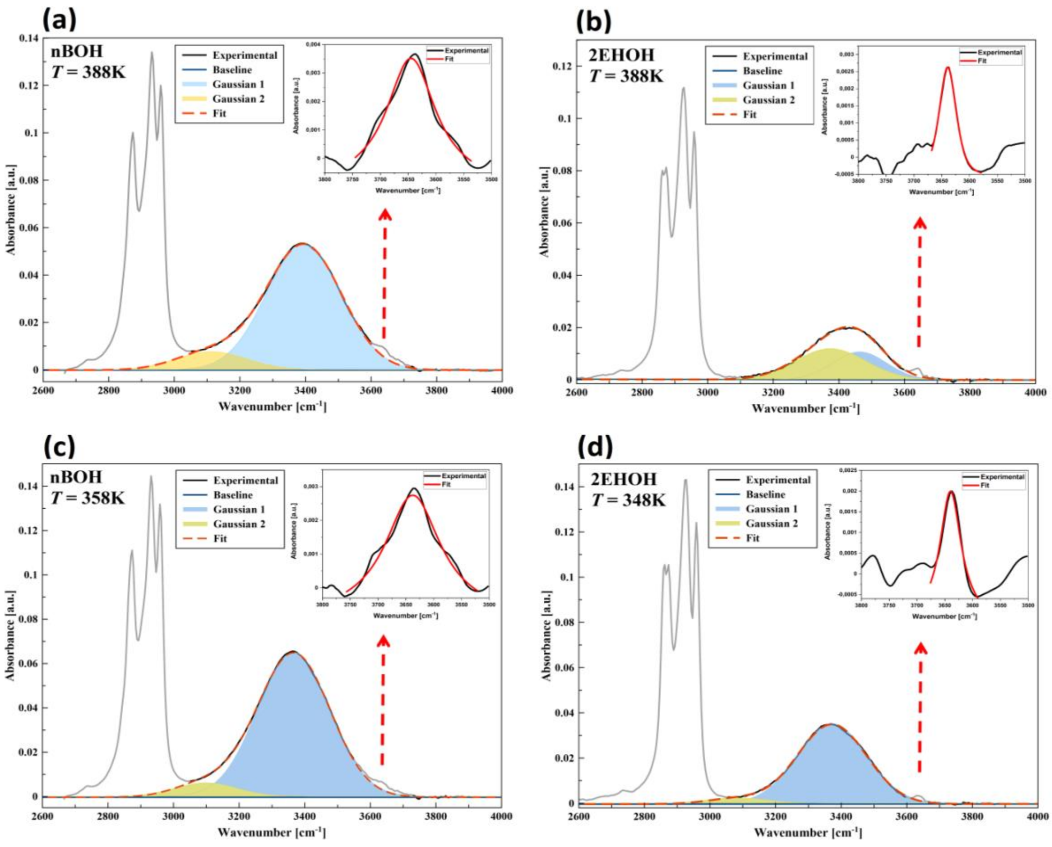

Figure S2. Decomposition of the ν_{OH} band of nBOH and 2EHOH in the frequency range of 2600-4000 cm^{-1} at *T* = 388 K (a,b), *T* = 358 K (c) and *T* = 348 K (d).

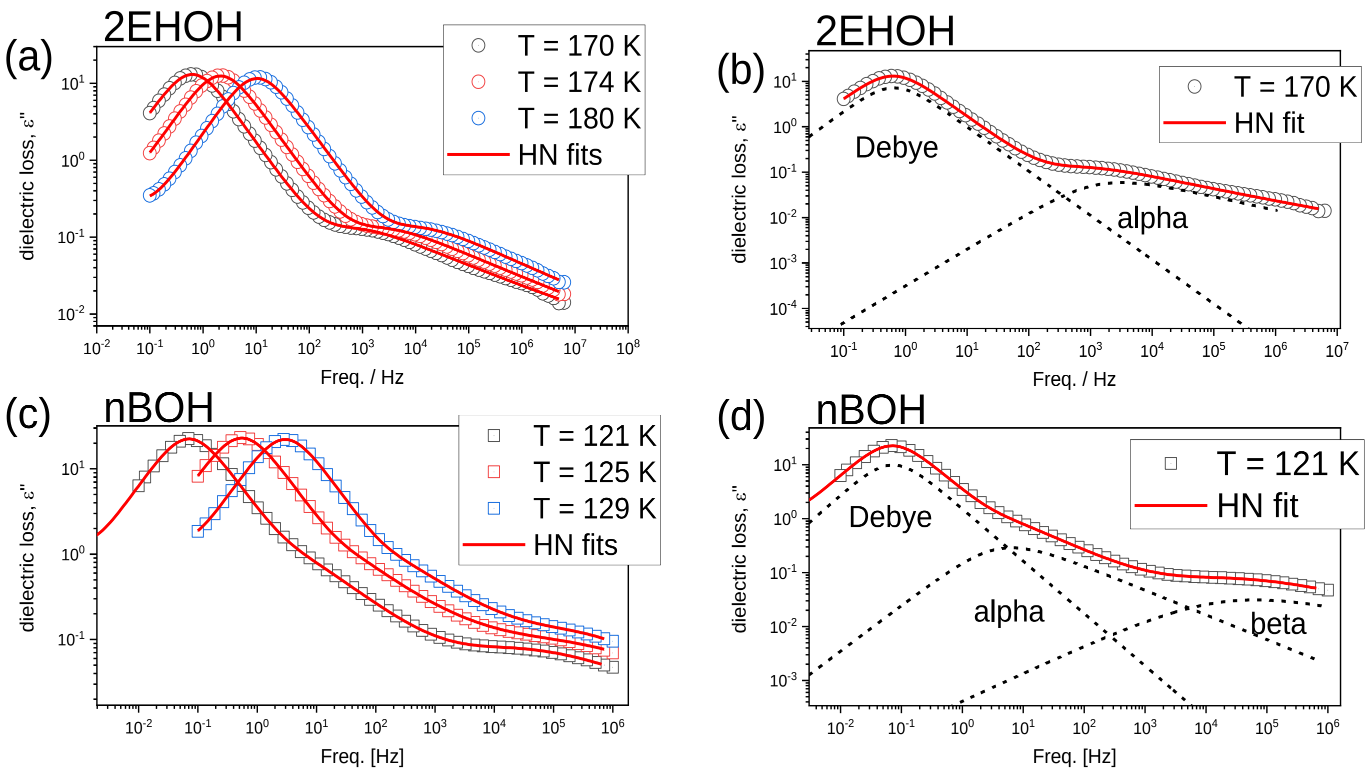

Figure S3. Selected dielectric loss spectra collected for 2EHOH (a,b) and nBOH (c,d) together with the best HN fits (solid red lines).

Table S1. HN fit parameters of both Debye and α-processes.

Temp. [K]	**Debye process**				**Alpha process**			
	α_{HN}	β_{HN}	$\Delta\varepsilon$	τ [s]	α_{HN}	β_{HN}	$\Delta\varepsilon$	τ [s]
**2EHOH**								
180	1	0.9703	23.78	0.01496	0.8219	0.3862	0.453	9.451E-6
174	1	0.9776	25.24	0.07545	0.8351	0.3953	0.401	3.863E-5
170	1	0.9747	26.05	0.2553	0.8578	0.3993	0.349	1.221E-4
**nBOH**								
129	1	0.9815	44.48	0.05368	0.9216	0.4912	1.41	3.569E-4
125	0.9958	0.98186	46.2	0.2889	0.9630	0.4933	1.205	0.00229
121	1	0.9709	45.32	2.246	0.8969	0.4976	1.441	0.01893

Table S2. VFT fit parameters of the α-process, glass transition temperatures determined from eq 2 and activation barriers of dissociation calculated according to eq 1.

Data set	log(τ_0 [s])	D_T	T_0 [K]	T_g [K] for τ_α = 100s	E_a [kJ/mol]
**2EHOH**					
Our data	-11.06	862.5	115.0	143.7	19.4±0.8
Data from ref 1	-12.04	1081.8	110.4	143.9	13.5±1.0
**nBOH**					
Our data	-10.86	730.4	86.8	111.4	5.3±0.4
Data from ref 2	-13.2	1000.9	86.0	114.7	-5.1±0.3

Table S3. Peak wavenumber, integrated intensity, and full-width at half maximum ($FWHM$) values obtained from the analysis of FTIR spectra for the alcohols under investigation, and estimated amounts of free OH bonds and the degree of OH association.

Material	**Peak wavenumber [cm^{-1}]**			
	***T*=298 K**		***T*=388 K**	
	H-bonded OH	free OH	H-bonded OH	free OH
nBOH	3318	---	3399	3629
2EHOH	3324	3634	3432	3640
	**Integrated intensity [arb. units]**			
nBOH	27.367	---	17.317	0.195
2EHOH	12.602	0.0152	5.053	0.082
	**Total integrated intensity [arb. units]**			
nBOH	27.358		17.512	
2EHOH	12.617		5.135	
	**Content of free OH bonds [%]**			
nBOH	0.00		1.11	
2EHOH	0.12		1.56	
	**Degree of OH association [%]**			
nBOH	100		98.89	
2EHOH	99.88		98.40	
	**Peak *FWHM* [cm^{-1}]**			
nBOH	242.95	---	270.91	64.85
2EHOH	217.66	19.26	243.79	28.62

References:

[1] Gainaru, C.; Kastner, S.; Mayr, F.; Lunkenheimer, P.; Schildmann, S.; Weber, H. J.; Hiller, W.; Loidl, A.; Bohmer, R. Hydrogen-bond Equilibria and Lifetimes in a Monohydroxy Alcohol. *Phys. Rev. Lett.* **2011**, *107*, 118304.

[2] Lederle, C.; Hiller, W.; Gainaru, C.; Bohmer, R. Diluting the Hydrogen Bonds in Viscous Solutions of n-butanol with n-bromobutane: II. A Comparison of Rotational and Translational Motions. *J. Chem. Phys*. **2011,** *134*, 064512.
